# Supplementary material for: Core Promoter Regions of Antisense and Long Intergenic Non-Coding RNAs
Source: Int J Mol Sci. 2023 May 3;24(9):8199. doi: 10.3390/ijms24098199 (PMC10179571; doi:10.3390/ijms24098199)
Supplement: Supplementary file 1 [file ijms-24-08199-s001.zip › ijms-2325021-supplementary/Table S1.pdf]

Table S1. Frequencies of occurrence of different octanucleotides in the positions *(-32 : -25)* of the full samples of *M.°musculus* and *H. sapiens*.

	M. musculus (-32 : -25)		*H. sapiens (-32 : -25)*	
1	TTTTTTTT	0.32%	GGTTTATC	0.43%
2	AAATAAAA	0.16%	GAGAATAA	0.34%
3	CTATAAAA	0.16%	GTTTTATA	0.26%
4	GACAATAT	0.16%	CTTTGTTT	0.21%
5	CTAGTTAT	0.13%	CCTATAAA	0.21%
6	CTATATAA	0.13%	TCTATAAA	0.17%
7	GGTTCATA	0.13%	GGGGCGGG	0.17%
8	GTTTAAAA	0.10%	TTATATAA	0.17%
9	CTATTTAT	0.10%	CGCGCCCC	0.13%
10	CCTCCCCA	0.10%	GGGAGAGA	0.13%
11	TATATAAG	0.10%	GCTTTTCT	0.13%
12	CCCGGCCC	0.10%	ATTCAAAA	0.13%
13	CCTATAAA	0.10%	GCCCCGCC	0.13%
14	AGGAGAAA	0.10%	CTCCCTTC	0.13%
15	GGGGCGAG	0.10%	CTCTTAAA	0.13%
16	CCGCCGCC	0.10%	CTATAAAA	0.13%
17	CTTTAAAA	0.10%	GCCATGCA	0.13%
18	GTATAAAT	0.10%	GGTTGAGG	0.13%
19	AGTCATAA	0.10%	GGGATCTG	0.13%
20	CCTCTCCC	0.10%	TATAAAAA	0.13%
