# Supplementary material for: Core Promoter Regions of Antisense and Long Intergenic Non-Coding RNAs
Source: Int J Mol Sci. 2023 May 3;24(9):8199. doi: 10.3390/ijms24098199 (PMC10179571; doi:10.3390/ijms24098199)
Supplement: Supplementary file 1 [file ijms-24-08199-s001.zip › ijms-2325021-supplementary/Table S2.pdf]

Table S2. Frequencies of occurrence (in percent's) of octanucleotides in the positions *(-32 : -25)* of the samples obtained imposing the condition *"**restricting the selection to promoters that contain a TATA box**"*.

	M. musculus (-32 : -25)		*H. sapiens (-32 : -25)*	
1	GACAATAT	1.61%	GAGAATAA	3.20%
*2	CTATAAAA	1.61%	GTTTTATA	2.40%
3	AAATAAAA	1.61%	CCTATAAA	2.00%
4	CTATATAA	1.29%	TTATATAA	1.60%
5	CCTATAAA	0.96%	TCTATAAA	1.60%
6	GTTTAAAA	0.96%	CTTTAAAA	1.20%
7	TATAAAAG	0.96%	TATAAAAA	1.20%
8	CTTAAAAG	0.96%	CTATAAAA	1.20%
9	CTATTTAT	0.96%	CCAATAAA	0.80%
10	GTATAAAT	0.96%	ATAAATAG	0.80%
11	CTTTAAAA	0.96%	CCCTTAAA	0.80%
12	TATATAAG	0.96%	GTATTTAT	0.80%
13	GTATAAAA	0.96%	CTTATAAA	0.80%
14	CAAATAAA	0.64%	GAATAAAT	0.80%
15	TATATAAA	0.64%	ATAAAGAC	0.80%
16	GCTATAAA	0.64%	TTACAAAA	0.80%
17	GCCATAAA	0.64%	TATAAATT	0.80%
18	AGATAAAA	0.64%	GCTTTAAA	0.80%
19	GGTATAAA	0.64%	TTTTAAAA	0.80%
20	GCTATTTA	0.64%	CTTAAAGG	0.80%
