# Supplementary material for: Core Promoter Regions of Antisense and Long Intergenic Non-Coding RNAs
Source: Int J Mol Sci. 2023 May 3;24(9):8199. doi: 10.3390/ijms24098199 (PMC10179571; doi:10.3390/ijms24098199)
Supplement: Supplementary file 1 [file ijms-24-08199-s001.zip › ijms-2325021-supplementary/Table S3.pdf]

Table S3. Frequencies of occurrence of different octanucleotides in the positions *(-30 : -23)* of the full samples of *M.°musculus* and *H. sapiens*.

	M. musculus (-30 : -23)		*H. sapiens (-30 : -23)*	
1	TTTTTTTT	0.39%	ACAATATA	1.61%
2	TATAAAAG	0.16%	CTATAAAA	1.29%
3	CAATATAA	0.16%	GTATAAAA	1.29%
4	AGTTATGT	0.13%	TTTAAAAG	1.29%
5	TATATAAG	0.13%	AATAAAAG	1.29%
6	TAAAAAGC	0.13%	CTATTTAG	0.96%
7	TAAGATCC	0.10%	TATAAATA	0.96%
8	AGAGAGAG	0.10%	TTTAAAAC	0.96%
9	TCCTTTTT	0.10%	TATATAAG	0.96%
10	TATAAAAA	0.10%	AATAAAAA	0.96%
11	ATAAAAGG	0.10%	TATTTATT	0.96%
12	GCCCCGCC	0.10%	GTATATAA	0.96%
13	ATAAAAAC	0.10%	AAATAAAT	0.96%
14	ATAAAAGC	0.10%	ATATAAGG	0.96%
15	GAAGTGGA	0.10%	TATAAAAG	0.96%
16	GGGAGGGG	0.10%	TTAAAAAG	0.64%
17	AAAAAAAA	0.10%	CTTAAAGA	0.64%
18	AATAAGGA	0.10%	TATAATAG	0.64%
19	CTGCTTCC	0.10%	ATAAAAAG	0.64%
20	ATAAAACC	0.10%	CTATAAAT	0.64%
