# Supplementary material for: Core Promoter Regions of Antisense and Long Intergenic Non-Coding RNAs
Source: Int J Mol Sci. 2023 May 3;24(9):8199. doi: 10.3390/ijms24098199 (PMC10179571; doi:10.3390/ijms24098199)
Supplement: Supplementary file 1 [file ijms-24-08199-s001.zip › ijms-2325021-supplementary/Table S4.pdf]

Table S4. Frequencies of occurrence of octanucleotides in the positions *(-30 : -23)* of the samples obtained imposing the condition *"**restricting the selection to promoters that contain a TATA box**"*.

	M. musculus (-30 : -23)		*H. sapiens (-30 : -23)*	
1	TATAAAAG	1.61%	GAATAAAA	4.00%
2	CAATATAA	1.61%	TTTATAAG	2.00%
3	TAAAAAGC	1.29%	ATAAAAGC	2.00%
4	TATATAAG	1.29%	ATATAAAG	1.60%
5	TATAAAAA	0.96%	CTTAAAAG	1.20%
6	ATAAAACC	0.96%	TATAAAGC	1.20%
7	ATAAAAAG	0.96%	TTTAAAAG	1.20%
8	ATAAAAGG	0.96%	AAATAAAA	1.20%
9	ATAAAAAC	0.96%	ATTTAAAA	1.20%
10	ATAAAAGC	0.96%	ATTTATAA	0.80%
11	TTAAAAGC	0.64%	ATATAAGG	0.80%
12	TTAAAGAG	0.64%	TATAAAAC	0.80%
13	TATTTAGT	0.64%	TTTAAATC	0.80%
14	TATAAATT	0.64%	AATAAAAA	0.80%
15	GATAAAAC	0.64%	TTTATATC	0.80%
16	ATAAAATG	0.64%	GTATAAAA	0.80%
17	CATAAATA	0.64%	TATATGAT	0.80%
18	AATAAAAG	0.64%	TTTATAAA	0.80%
19	AATAAATG	0.64%	ACATAAAA	0.80%
20	TTATAAAG	0.64%	AAATAGAA	0.80%
