# Supplementary material for: Core Promoter Regions of Antisense and Long Intergenic Non-Coding RNAs
Source: Int J Mol Sci. 2023 May 3;24(9):8199. doi: 10.3390/ijms24098199 (PMC10179571; doi:10.3390/ijms24098199)
Supplement: Supplementary file 1 [file ijms-24-08199-s001.zip › ijms-2325021-supplementary/Table S5.pdf]

Table S5. Frequencies of occurrence of different octanucleotides in the positions *(-29 : -22)* of the full samples of *M. musculus* and *H. sapiens*.

	M. musculus (-29 : -22)		*H. sapiens (-29 : -22)*	
1	TTTTTTTT	0.45%	AATAAAAG	0.64%
2	AATATAAG	0.16%	TTATAAGG	0.26%
3	GAGAGAGA	0.13%	TTATCAGG	0.26%
4	GTTATGTT	0.13%	TGTTTACC	0.21%
5	TAAAACCC	0.13%	ATAAAAAC	0.13%
6	GGTAAAGT	0.13%	GGCGGGGC	0.13%
7	AAGATCCT	0.10%	CCTCTCCC	0.13%
8	TAAAAACC	0.10%	CGTCCCGC	0.13%
9	TTGTTTTT	0.10%	CCGCCCCC	0.13%
10	GGCGTGGC	0.10%	GGAAGAGG	0.13%
11	TAAAGAGG	0.10%	CCTCCTCC	0.13%
12	ATAAAAAG	0.10%	TAAAAGCT	0.13%
13	CCCCGCCC	0.10%	ATGCAGGG	0.13%
14	TAAAAGCA	0.10%	CTTGGCCA	0.13%
15	TATAAAGC	0.10%	TTAAAAGG	0.13%
16	GGGAGGGG	0.10%	ATTTTTTT	0.13%
17	ATAAAAGA	0.10%	GCTAATAA	0.13%
18	ATAAAAGG	0.10%	TGAGGTGG	0.13%
19	CTGCCCGC	0.10%	TATAAAGC	0.13%
20	AGAAAAGG	0.10%	TTATCAAG	0.13%
