# Supplementary material for: Core Promoter Regions of Antisense and Long Intergenic Non-Coding RNAs
Source: Int J Mol Sci. 2023 May 3;24(9):8199. doi: 10.3390/ijms24098199 (PMC10179571; doi:10.3390/ijms24098199)
Supplement: Supplementary file 1 [file ijms-24-08199-s001.zip › ijms-2325021-supplementary/Table S6.pdf]

Table S6. Frequencies of occurrence of octanucleotides in the positions *(-29 : -22)* of the samples obtained imposing the condition *"**restricting the selection to promoters that contain a TATA box**"*.

	M. musculus (-29 : -22)		*H. sapiens (-29 : -22)*	
1	AATATAAG	1.61%	AATAAAAG	6.00%
2	TAAAACCC	1.29%	TTATAAGG	2.40%
3	ATAAAAGG	0.96%	TTAAAAGG	1.20%
4	ATAAAAGA	0.96%	ATAAAGCT	1.20%
5	TAAAAACC	0.96%	ATAAAAAC	1.20%
6	ATAAATAG	0.96%	TTTAAAAG	1.20%
7	ATAAAATG	0.96%	TATAAAGC	1.20%
8	ATAAAAAG	0.96%	ATTTAAGA	0.80%
9	TAAAGAGG	0.96%	CTTTATAA	0.80%
10	TAAAAAGG	0.96%	TTATATCC	0.80%
11	TATAAAGC	0.96%	ATAAATAC	0.80%
12	TAAAAGGA	0.96%	TAAAACCC	0.80%
13	ATAAAAGC	0.96%	ATATGATC	0.80%
14	TAAAAGCA	0.96%	TTTAAAAA	0.80%
15	TTAAAAAC	0.64%	AGAAAAGG	0.80%
16	AAAAAGCC	0.64%	TTAAAAGC	0.80%
17	ATAAATTG	0.64%	TAAAAGCA	0.80%
18	TATTTAAG	0.64%	TATAAAAG	0.80%
19	TATAAAGT	0.64%	TTATAAAG	0.80%
20	ATAAAACC	0.64%	CATAAAAG	0.80%
