# Supplementary material for: Core Promoter Regions of Antisense and Long Intergenic Non-Coding RNAs
Source: Int J Mol Sci. 2023 May 3;24(9):8199. doi: 10.3390/ijms24098199 (PMC10179571; doi:10.3390/ijms24098199)
Supplement: Supplementary file 1 [file ijms-24-08199-s001.zip › ijms-2325021-supplementary/Table S8.pdf]

Table S8. Frequencies of occurrence of octanucleotides in the positions *(-28 : -21)* of the samples obtained imposing the condition *"**restricting the selection to promoters that contain a TATA box**"*.

	M. musculus (-28 : -21)		*H. sapiens (-28 : -21)*	
1	ATATAAGG	1.61%	ATAAAAGC	6.40%
2	TAAAAGGA	0.96%	TATAAGGG	2.00%
3	ATAAAGCC	0.96%	TAAAGCTG	1.20%
4	AAAAGGAA	0.96%	TTTATAAG	1.20%
5	AAAAAGCT	0.64%	ATAAAAGA	1.20%
6	AAAGAGGG	0.64%	TTAAAAGT	1.20%
7	TAAAAGAG	0.64%	TATATAAA	0.80%
8	AAAACCCA	0.64%	TAAAAGGG	0.80%
9	AAAAGCCA	0.64%	TAAAAACT	0.80%
10	TAAAGCTA	0.64%	TATGATCA	0.80%
11	ATTTAAGC	0.64%	AAAAGAAA	0.80%
12	AAAAAGGA	0.64%	ATAAAGGG	0.80%
13	ATAAAGTG	0.64%	TAAAAGGA	0.80%
14	TAAAACCA	0.64%	ATAAAAAC	0.80%
15	TAAGAGGA	0.64%	TAAAAACC	0.80%
16	TAAATTGC	0.64%	ACAAAGGC	0.80%
17	AAAAGGGG	0.64%	TTAAAGGG	0.80%
18	TAAAACTC	0.64%	TATTTAAA	0.40%
19	ATAAGAGG	0.64%	AAAAATCT	0.40%
20	ATTAATAG	0.64%	TATATAGA	0.40%
