# Supplementary material for: Core Promoter Regions of Antisense and Long Intergenic Non-Coding RNAs
Source: Int J Mol Sci. 2023 May 3;24(9):8199. doi: 10.3390/ijms24098199 (PMC10179571; doi:10.3390/ijms24098199)
Supplement: Supplementary file 1 [file ijms-24-08199-s001.zip › ijms-2325021-supplementary/Table S9.pdf]

Table S9. Frequencies of occurrence of dinucleotides in the (−2, +2) positions of INR.

M. musculus		*H. sapiens*	
TCAG	7.99%	TCAG	5.81%
CCAG	7.51%	CCAG	5.52%
GCAG	5.26%	GCAG	3.85%
TCAC	4.29%	TCAC	3.33%
CCAC	3.18%	CCAC	3.25%
ACAG	2.89%	CTGG	2.74%
TTAG	2.76%	ACAG	2.22%
CCAT	1.95%	TCAT	2.09%
GCAC	1.95%	CTGA	2.09%
CTGA	1.92%	GCGG	2.09%
CTGT	1.85%	CCGG	2.05%
TTGT	1.75%	TTGG	2.01%
TCAT	1.66%	CTGT	1.92%
TTCT	1.56%	CCGC	1.75%
CTGC	1.56%	GTGG	1.71%
CTAG	1.53%	TTAG	1.67%
TCCT	1.49%	GCAC	1.62%
CCGC	1.14%	TCCT	1.62%
TCAA	1.14%	CTAG	1.58%
CTAA	1.14%	TTGT	1.50%
