# Supplementary material for: Core Promoter Regions of Antisense and Long Intergenic Non-Coding RNAs
Source: Int J Mol Sci. 2023 May 3;24(9):8199. doi: 10.3390/ijms24098199 (PMC10179571; doi:10.3390/ijms24098199)
Supplement: Supplementary file 1 [file ijms-24-08199-s001.zip › ijms-2325021-supplementary/TableS 7.pdf]

Table S7. Frequencies of occurrence of different octanucleotides in the positions *(-28 : -21)* of the full samples of *M. musculus* and *H. sapiens*.

	M. musculus (-28 : -21)		*H. sapiens (-28 : -21)*	
1	TTTTTTTT	0.45%	ATAAAAGC	0.68%
2	ATATAAGG	0.16%	TTTTTTTT	0.26%
3	AGATCCTC	0.13%	TATAAGGG	0.21%
4	TTATGTTC	0.13%	TATCAGGG	0.21%
5	ATTAATAG	0.10%	GTTTACCT	0.21%
6	GGGGGTGG	0.10%	GGCGCGGG	0.13%
7	TAAAAGGA	0.10%	GAGAGGGA	0.13%
8	CAAAACCC	0.10%	GGGGCGGG	0.13%
9	ATAAAGCC	0.10%	AAAAGAAA	0.13%
10	CTGAGCTC	0.10%	GGAGGGAG	0.13%
11	CCAGGAGG	0.10%	GGAAGCGC	0.13%
12	GAAAAGGA	0.10%	TTTATAAG	0.13%
13	AAAAGGAA	0.10%	TGCAGGGC	0.13%
14	TGAAGGGA	0.10%	CTTGAGCT	0.13%
15	AAAAGCCA	0.10%	TTGGCCAG	0.13%
16	TAAATACC	0.10%	ATAAAAGA	0.13%
17	TAAGAGGA	0.10%	TAAAAGGA	0.13%
18	AAAACCCC	0.10%	CTAATAAT	0.13%
19	GGGAGGGG	0.10%	CCTCCCCC	0.13%
20	TTAGGAGG	0.10%	TTAAAAGT	0.13%
